# Rift Valley fever virus structural proteins: expression, characterization and assembly of recombinant proteins

**DOI:** 10.1186/1743-422X-5-82

**Published:** 2008-07-18

**Authors:** Li Liu, Cristina CP Celma, Polly Roy

**Affiliations:** 1 Department of Infectious and Tropical Diseases, London School of Hygiene and Tropical Medicine, Keppel Street, London, WC1E 7HT, UK; 2 Present address: Centre for Infectious Disease, Institute of Cell and Molecular Science, Barts and The London, Queen Mary's School of Medicine and Dentistry, The Blizard Building, 4 Newark Street, London, E1 2AT, UK

## Abstract

**Background:**

Studies on Rift Valley Fever Virus (RVFV) infection process and morphogenesis have been hampered due to the biosafety conditions required to handle this virus, making alternative systems such as recombinant virus-like particles, that may facilitate understanding of these processes are highly desirable. In this report we present the expression and characterization of RVFV structural proteins N, Gn and Gc and demonstrate the efficient generation of RVFV virus-like particles (VLPs) using a baculovirus expression system.

**Results:**

A recombinant baculovirus, expressing nucleocapsid (N) protein of RVFV at high level under the control of the polyhedrin promoter was generated. Gel filtration analysis indicated that expressed N protein could form complex multimers. Further, N protein complex when visualized by electron microscopy (EM) exhibited particulate, nucleocapsid like-particles (NLPs). Subsequently, a single recombinant virus was generated that expressed the RVFV glycoproteins (Gn/Gc) together with the N protein using a dual baculovirus vector. Both the Gn and Gc glycoproteins were detected not only in the cytoplasm but also on the cell surface of infected cells. Moreover, expression of the Gn/Gc in insect cells was able to induce cell-cell fusion after a low pH shift indicating the retention of their functional characteristics. In addition, assembly of these three structural proteins into VLPs was identified by purification of cells' supernatant through potassium tartrate-glycerol gradient centrifugation followed by EM analysis. The purified particles exhibited enveloped structures that were similar to the structures of the wild-type RVFV virion particle. In parallel, a second recombinant virus was constructed that expressed only Gc protein together with N protein. This dual recombinant virus also generated VLPs with clear spiky structures, but appeared to be more pleomorphic than the VLPs with both glycoproteins, suggesting that Gc and probably also Gn interacts with N protein complex independent of each other.

**Conclusion:**

Our results suggest that baculovirus expression system has enormous potential to produce large amount of VLPs that may be used both for fundamental and applied research of RVFV.

## Background

RVFV is a member of the Phlebovirus genus within the *Bunyaviridae *family. It is endemic in North Africa and the Arabia peninsula, infecting both livestock and humans [1,2]. Infection of humans provokes a wide range of symptoms, from fever to fatal encephalitis, retinitis and hepatitis associated with haemorrhages [3,4] while in livestock and wild ruminants it causes teratogeny and abortion in pregnant animals and produces high rate of mortality in young animals. Like other members of the genus, RVFV is vector-borne, mainly transmitted by mosquitoes of *Aedes *species, although many others species are also capable of virus replication and transmission and thus increasing the possibilities of outbreaks in Sub-Saharan regions [5,6].

RVFV is an enveloped virus with a diameter of 90 to 110 nm and a core element of 80 to 85 nm [7,8]. The viral genome consists of single-stranded, tripartite RNA, among which the large (L) and medium (M) segments are negative polarity, and the small (S) segment is ambisense polarity [9-11]. The L segment codes for the RNA-dependent RNA polymerase, which is packed together with the genomic RNA segments within the virus particles [9]. The S segment codes for two proteins, the structural nucleoprotein (N) in the negative sense and the small non-structural protein (NSs) in the positive sense [10]. The N protein is the nucleocapsid protein and is closely associated with the genome RNA in the virion particles, and the NSs protein inhibits host gene transcription in the infected cells thereby blocking interferon production [12,13]. The M segment encodes two structural glycoproteins Gn (encoded by amino-terminal sequences) and Gc (encoded by carboxy-terminal sequences), and two non-structural proteins the 78 kDa and the 14 kDa NSm protein [11,14,15] that are produced in a complex strategy of translation initiation and polyprotein processing. The mRNA transcribed from the M segment has five in-frame initiation codons upstream of the Gn and Gc sequence [14-16]. The 78-KDa protein is translated from the first AUG and includes the entire coding sequence of Gn whereas NSm protein starts from the second AUG to the beginning of Gc. Neither the 78-KDa nor the 14 KDa proteins seems to be essential for virus replication in cell culture [16,17], and their function is still unclear.

The structural glycoproteins Gn and Gc are expressed as a polyprotein precursor that is processed by cellular proteases during its maturation and result in a heterodimeric complex [16]. It has been shown that oligomerization of viral glycoproteins occurs most probably in the endoplasmic reticulum (ER) and is critical for their transit to the Golgi apparatus [16]. As for other members of the *Bunyaviridae *family, RVFV glycoproteins are localized to the Golgi apparatus [18,19] where the remaining structural proteins and the genome are recruited prior to budding. Although the receptor utilized by RVFV is still unknown, Gn and Gc are sufficient for virus entry during infection and a low pH activation after endocytosis of the virion is essential for this process [20,21].

Studies on RVFV infection process and morphogenesis have been hampered due to the requirement of high biosafety conditions to handle this virus, thus alternative systems that may facilitate understanding of these processes are highly desirable. To this end a number of recombinant protein expression systems including bacteria, vaccinia virus, baculovirus systems and more recently alphavirus-based vector have been used to generate RVFV structural proteins [22-25]. However, to date production of multi-component RVFV VLPs has not been achieved. Assembly of VLPs of many viruses by recombinant expression systems had been highly successful both for understanding the fundamental aspects of virus life cycle as well as for its immunogenic properties (see reviews [26,27]). In this report we present the expression and characterization of RVFV structural proteins N, Gn and Gc and demonstrate the efficient generation of VLPs in insect cells using a single recombinant baculovirus.

## Results

### Expression of N protein produces complex structures

**Figure 1:**
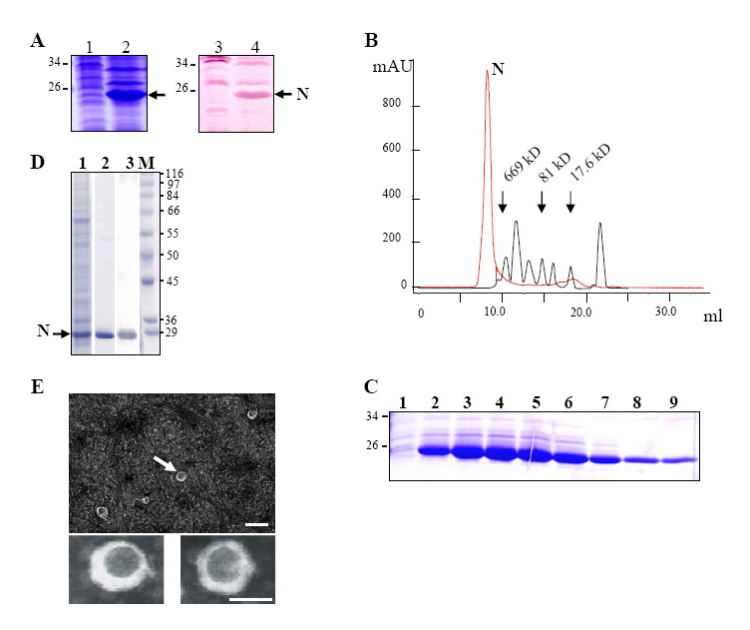
**Expression and purification of RVFV nucleoprotein (N) protein**. Insect *Sf*9 cells were infected with a recombinant baculovirus expressing RVFV N protein and four days after infection the expression of N was assessed. **A) **Infected cell lysate expressing N protein was analyzed by SDS-PAGE followed by Commassie Brilliant blue staining (lane 2) or Western blotting (lane 4) and compared with total proteins from uninfected insect cells (lanes 1 & 3). Protein markers were included and sizes in kilo-Dalton (kDa) are shown at the right. **B) **Purification of N protein by gel filtration. The position of the peak correspondent to N and the relative elution position of molecular markers are indicated. **C) **Samples of the gel filtration fractions corresponding to the peak of protein were analyzed by SDS-PAGE and stained with Commassie brilliant blue (lanes 2 to 9). An aliquot of a fraction, prior to N protein fraction, was included as a control (lane1). The relative position of molecular marker is indicated in KDa. **D) **Purified N protein was analyzed by SDS-PAGE followed by Commassie Brilliant blue staining (lane 2) or Western blotting (lane 3) and compared with total proteins from infected insect cells (lane 1). Protein markers were included (lane M) and sizes in KDa are shown at the right. **E) **An aliquot of purified N protein were negatively stained with 3% phosphotungstic acid (PTA), pH 6.8 and visualized by electron microscopy. A particulate structure is indicated with an arrow in upper panel and lower panel shows amplified particles. Bar represents 100 nm.

The nucleoprotein N is the most abundant viral component in the RVFV virion and also in virus infected cells. N is tightly associated with the three genomic RNA segments, forming the three nucleocapsids. N protein plays a number of roles that are essential in virus replication. In addition it also interacts with L, Gn and Gc, although the nature of their interactions have not yet been defined. In order to generate N protein in sufficient amount in the absence of other viral proteins we generated a recombinant baculovirus (as described in Methods) and examined the level of N protein expression in insect cells. Insect *Sf*9 cells were infected with this recombinant baculovirus for four days and the presence of N protein in the cell lysate was assessed by SDS-PAGE analysis. A strong extra band of 26 KDa equivalent to the expected size of the N protein was detected in the infected cell lysate (Fig. 1A, lane 2). This band was not present in the lysate from uninfected cells (Fig. 1A, lane 1). Western blot analysis using monoclonal antibody specific to RVFV N protein confirmed that the extra band was the RVFV N protein (Fig. 1A, lane 4).

Recent studies have demonstrated that the basic oligomeric status of N protein in purified ribonucleoprotein (RNP) from RVFV infected cells is a dimer, however it exhibited multimeric organization when RNPs were cross-linked with glutaraldehyde [12]. To investigate if recombinant N synthesized in insect cells is capable of oligomerisation, the supernatant of infected insect cells were clarified, ultracentrifuged through a sucrose cushion and protein products were analyzed by gel filtration column chromatography. The products obtained from the gel filtration are shown in Fig. 1B. A distinct protein peak was detected in the exclusion region (the column exclusion size limit was 1300 kD) suggesting that the N protein was able to form complex structures. To determine the position of N protein complex a series of protein control molecular markers were included and their relative position is indicated in the figure. When aliquots of gel filtration fractions were analyzed by SDS-PAGE (Fig. 1C), a band of the expected size for N was detected, suggesting that N was the major component in those fractions. To confirm further that the eluted band was indeed the N protein of RVFV, and had the same mobility with the N protein band of the cell lysate an aliquot was analyzed by Western blot using anti-N antibody (Fig. 1D, lane 3).

To determine if N protein containing fractions could form any particulate complex structure, fractions containing N protein were clarified by ultracentrifugation and aliquots were visualized by electron microscopy (EM). Distinct particulate structures could be detected under EM (Fig. 1E). The size of these structures ranged from 56 to 78 nm, suggesting that N could indeed form complex multimeric structures.

### Expression of three structural proteins by a single recombinant virus

RVFV virus particle are enveloped, and the two structural glycoproteins Gn and Gc are inserted in the membrane that surrounds the RNP. To investigate if RVFV glycoproteins can be assembled together with N protein in baculovirus expression system, a dual protein expression vector was designed. Previous works using vaccinia and baculovirus systems have shown that the expression of Gn/Gc from the fourth AUG of M segment produce high level and correct processing of both proteins [24,28]. Indeed of the five AUG initiation codons present in the upstream sequence of Gn only the fourth AUG is in optimal translation context sequence. Therefore for the baculovirus construct, the open reading frame of M segment from the fourth AUG was used. The Gn/Gc and N sequences were inserted into the baculovirus transfer vector under the control of two separate polyhedrin promoters. The recombinant baculovirus was generated as described in Methods.

**Figure 2:**
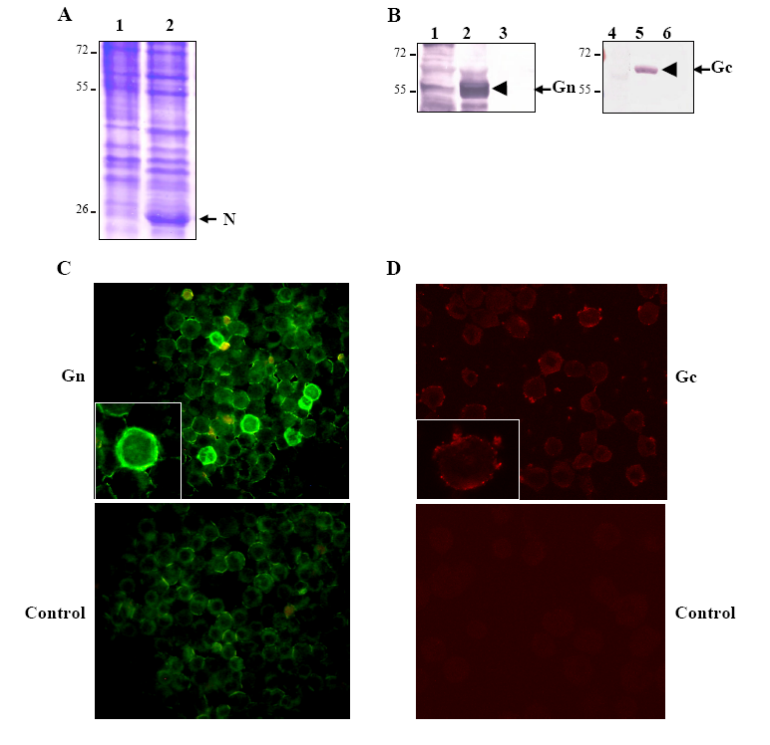
**Detection of RVFV Gn and Gc and the cell surface expression**. **A) **Cell lysate from infected cells with a recombinant baculovirus expressing RVFV N, Gn and Gc were analyzed by SDS-PAGE followed by Commassie blue stain (lane2). As a control cell lysate from uninfected cells were included (lane 1). **B) **Western blot using specific antibodies against Gn (lane 2) or Gc (lane 5) was performed with cell lysate expressing RVFV proteins N, Gn and Gc. As a control cell lysates from uninfected cells (lanes 1 and 4) or expressing RVFV N protein (lanes 3 and 6) were included. **C) **Cell surface expression of RVFV Gn. Infected cells expressing RVFV N, Gn and Gc proteins were fixed and processed for immunoflorescence under non-permeabilizing conditions. To detect RVFV Gn protein, a specific antibody was used followed by an anti-mouse-FITC conjugated secondary antibody (upper panel). As control cells expressing β-Gal protein were processed similarly (lower panel). **D) **Cell surface expression of RVFV Gc. Cells expressing RVFV N, Gn and Gc were examined for cell surface expression of Gc using a specific antibody against Gc and a anti mouse-TRITC as secondary antibody (upper panel). Control cells were included (lower panel).

Insect cells were infected with the recombinant baculovirus containing the three RVFV genes. After 3 days cells were lysed and the lysates were analyzed by SDS-PAGE followed by Commassie blue staining. While expression of N protein was at a high level and clearly visible, bands of Gc and Gn were not convincing (Fig. 2A, lane 2). Therefore a Western analysis using the appropriate antibodies was performed. An aliquot of cell lysate from uninfected cells and from baculovirus infected cells expressing β-Gal were also included as control. Only in samples from cells infected with recombinant baculovirus, proteins bands corresponding to Gn (Fig. 2B, lane 2) and Gc (Fig. 2B, lane 5) could be detected by specific antibodies against those proteins. This result confirmed that in addition to N protein, both Gn and Gc were also expressed and the Gc/Gn was properly processed to generate the proteins in the insect cells.

### Gn and Gc are targeted to the plasma membrane of insect cells

It has been reported previously that when RVFV Gn and Gc are expressed individually, Gn is targeted to the Golgi while Gc is retained in the ER [18,19]. However, Filone *et al*. have recently shown that the overexpression of Gn and Gc by alphavirus replicon vectors resulted in the localization of these proteins in the cell surface [20]. Therefore it was of interest if this effect could be observed in insect cells by recombinant baculovirus expressing these glycoproteins. To visualize the expression of Gn/Gc complex on the cell surface, insect cells were infected with the recombinant baculovirus expressing Gn, Gc and N proteins and 30 hours post-infected cells were fixed and processed for immunofluorescence. Since these cells were not permeabilized only proteins expressed in the surface of cells should be detected. When specific antibody against Gn was used as a primary antibody and FITC-conjugated as secondary antibody, a strong signal around the surface of infected cells was easily visible (Fig. 2C, upper panel). Similar result was obtained when a specific antibody against Gc and TRITC-conjugated secondary antibody were used (Fig. 2D, upper right panel). As a control, cells were infected with a recombinant baculovirus expressing β-Galactosidase protein and processed similarly. Although low level of background was detected when the FITC-conjugated secondary antibody was used, no background was observed for the TRITC-conjugated antibody (Fig. 2C and 2D, lower panels).

Thus, the expression of RVFV Gn and Gc proteins in insect cells resulted in detection of both proteins on the surface of the non-permeabilized infected cells. The presence of Gn and Gc on the surface of infected insect cell suggests that the both proteins were correctly folded and properly processed.

### Surface expression of RVFV Gn/Gc can induce membrane fusion

**Figure 3:**
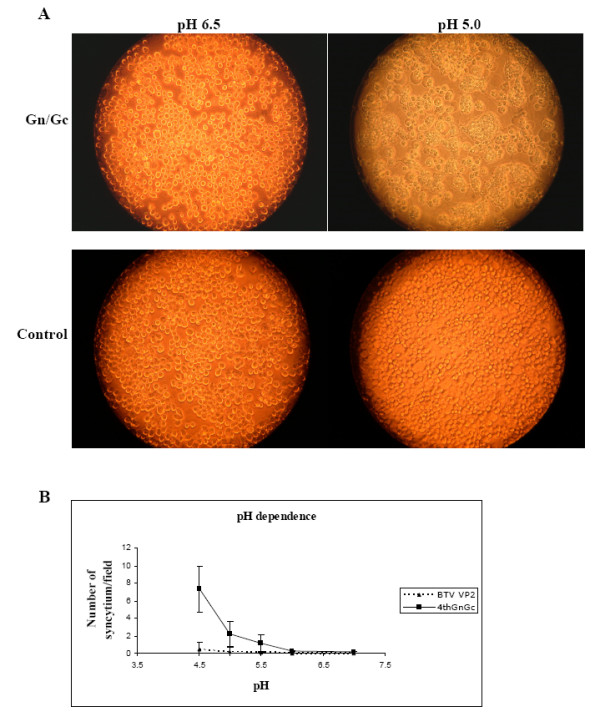
**Fusogenic activity of RVFV Gn and Gc proteins**. **A) **Insect cells were infected with a baculovirus expressing RVFV N, Gn and Gc for 24 hours and a monoclonal antibody against baculovirus gp64 were added to the media. After 2 hours the media was replaced with low pH media (pH 5.0) for two minutes and then replaced with normal media (right, upper panel). As controls, infected cells expressing RVFV proteins were kept at normal pH media of 6.5 (left, upper panel). As negative control, infected insect cells expressing BTV VP2 protein were included and pH shift was performed (right, lower panel) or the media was kept at neutral pH (left, lower panel). Pictures were taken at 200× magnification. **B) **Quantification of fusion capacity. The number of syncytia per field was counted by visual microscopy at 400× magnification and the average and standard deviation were calculated. Each assay was performed in triplicate.

It has been demonstrated that Gn and Gc are responsible for virus entry during natural infection using a class II fusion mechanism activated by low pH [21,29]. More recently the cell-cell fusion activity was demonstrated for Gn and Gc proteins that were expressed on the surface of cells at high levels using alphavirus replicon vectors [20]. Therefore to determine if recombinant Gn and Gc proteins expressed in insect cells were functionally active, fusion of adjacent membranes was investigated. *Sf*9 cells were infected with the baculovirus expressing both Gn and Gc proteins as described above and 24 h post-infected cells were incubated for two hours with a monoclonal antibody against gp64, a baculovirus surface glycoprotein, in order to inhibit activity of gp64 that has ability to induce cell-cell fusion after low pH induction. Cell media were then shift to pH 5.0 for two minutes and regularly examined for syncytia formation. Large syncytia were observed in cells expressing Gn/Gc proteins after two hours of treatment at low pH but no evidence of fusion was detected in cells maintained at normal pH of 6.5 (compare Fig. 3A, upper panels). As a control cells infected with another recombinant baculovirus that expresses Bluetongue virus (BTV) outer capsid protein VP2 [30], a non-fusogenic protein was also included. No evidence of syncytia formation was observed in VP2 expressed control cells (Fig. 3A, lower panels). These results suggest that Gn/Gc complex was functionally active and was solely responsible for inducing adjacent membrane fusion after low pH treatment.

To further characterize the pH dependence of the fusion activity of the complex Gn/Gc a range of pH were tested. The fusogenic ability was assessed as the average number of syncytia in at least 20 fields of visual microscopy at 100× magnification, in three independent experiments. As shown in Fig. 3B a significant number of syncytia were counted when cells expressing Gn/Gc were treated at low pH (between pH 4.5 to 5.5) and as expected the number decrease at high pH (pH 6.0 to 7.0). As expected, no evidence of fusion was observed in control cells expressing BTV VP2. These results demonstrated that Gn/Gc expressed in insect cells by recombinant baculovirus has a pH dependent fusion activity. Similar result were observed with alphavirus expressed Gn/Gc [20].

These results suggest that Gn/Gc expressed in the baculovirus expression system is fully functional and share similar characteristics with that of native RVFV infection and other recombinant systems.

### Co-expression of RVFV N and Gn/Gc or GC protein assemble into virus-like particle

**Figure 4:**
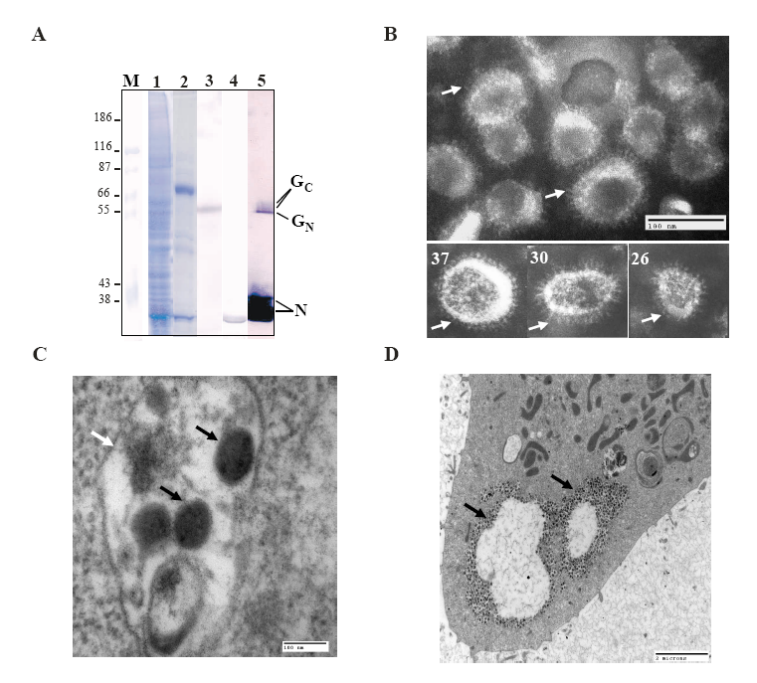
**Expression of N, Gn and Gc proteins produced virus-like particles**. **A) ***Sf*9 cells were infected with the recombinant baculovirus expressing RVFV N, Gn/Gc proteins and after 4 days both infected cells and the media were harvested. An aliquot from the infected cell lysate was analyzed by SDS-PAGE and stained by Commassie Brilliant blue (lane1). The media was clarified followed by ultracentrifugation on a potassium tartrate-glycerol gradient. An aliquot of purified material was analyzed as before (lane 2). Confirmation of viral proteins in purified samples was performed by Western blotting using monoclonal antibodies against either Gn (lane3) or N (lane 4) or with a polyclonal antibody against RVFV Zinga strain (lane 5). Protein markers were included (lane M) and sizes in kDa are shown on the right. **B) **Negative staining of purified VLPs. The spiky structures of the particle surface units consisting of glycoproteins Gn and Gc are indicated by arrows (upper panel). The spiky surface units are indicated by arrows (lower panels). The number of the surface unit of each particle is indicated at the upper left corner. Bar represents 100 nm. **C) **EM of infected cells' section showing VLPs are released into vacuoles. Note the presence of particles (black arrow) within the membrane (white arrow) of the vacuole boundaries. **D) **Same showing virus inclusion body in the cytoplasm indicated by arrows.

Since the recombinant N protein alone could initiate assembly of a particulate structure in insect cells it was likely that the expression of Gn and Gc together with the N protein may assemble as a particulate structure. To examine if the three expressed proteins could assemble into VLP, the supernatant from infected cells were collected after three days of infection. After clarification, the supernatant was loaded on to a 20% sucrose cushion and subjected to ultracentrifugation. The pellet was subsequently resuspended and further purified by ultracentrifugation through potassium tartrate-glycerol gradient and fractions were collected. Aliquots were analyzed by SDS-PAGE (data not shown) and those fractions with a band corresponding to N were concentrated by ultracentrifugation. The presence of Gn and N in the concentrated sample was detected by Western blot using monoclonal antibodies (Fig. 4A, lane 3 and 4), and all three proteins, N, Gn and Gc were also detected by polyclonal antibody against RVFV virus particles (Fig. 4A, lane 5).

In order to analyze if this concentrated sample indeed contained VLPs, an aliquot of the purified and concentrated fraction was examined by EM. Particulate structures with a spiky outer layer ranging from 90–120 nm were found in this fraction (Fig. 4B). These structures resemble the structure of RVFV. Some particles preserved nearly perfect surface subunits, which were presumably formed by Gn and Gc heterodimeric complex similar to that of virion particles [8] (Fig. 4B). The clarity of these surface spikes could easily be counted around 26 to 37 as shown in Fig. 4B (note the three particles in the lower panels). These results suggest that the structures purified from the supernatant of cells expressing RVFV structural proteins N and Gn/Gc are indeed VLPs.

Further, to determine the localization of VLPs in the cytosol and to confirm that VLPs were matured in the vacuoles, insect cells infected with the recombinant baculovirus expressing the three RVFV proteins were harvested, fixed and processed for ultra-section analysis. The results obtained from EM analysis showed that particulate structures, similar to RVFV virion particles, were released into vacuoles (Fig. 4C, indicated by black arrows). There were also a large number of inclusion bodies accumulated in the cytoplasm (Fig. 4D, indicated by arrow). Similar virion particles and inclusion bodies have also been reported to be present in RVFV-infected hepatocytes [7].

**Figure 5:**
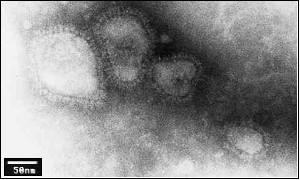
**Assembly of VLPs by expression of RVFV N and Gc proteins**. The supernatant of cells expressing N and Gc was purified as described in Methods and a sample of the purified material was stained and analyzed by EM. VLP structures with variable shapes and sizes were detected.

Whether both Gn and Gc were essential to form the VLPs was further investigated by expressing only Gc protein, together with N protein. In this construct, we used the same strategy as above except that an extra base was introduced in to the M sequence to create a frame shift in the Gn sequence. As a result, the translation of Gn was terminated after 47 amino acids. After 3 days of infection with the recombinant baculovirus expressing Gc and N, the supernatant was collected. After clarification, the supernatant was further purified by potassium tartrate-glycerol gradient and a visible band was collected. When the purified material from gradient was analyzed by EM a significant number of VLPs with spiky structures on the surface were identified (Fig. 5). These particles exhibited different morphology than those formed by either N protein alone or the VLPs with all three proteins. These Gc/N particles had spiky structure on the surface typical of a membrane glycoprotein but were much more pleomorphic than the VLPs consisted of both glycoproteins (Fig. 5).

The results suggest that Gc was expressed and together with N protein produced spiky virus-like structures. Therefore, it can be hypothesized that Gc (and probably Gn) interact with RNP complex independent of each other during virus infection.

## Discussion

RVFV is an important pathogen which infects both humans and livestock with a mortality rate of 1–3% among humans. Studies on the assembly of RVFV are particularly difficult due to the level of biosafety facilities necessary to undertake these studies. For this reason the development of alternative models with lower biosafety requirements is crucial for this virus.

In this work we present evidence of VLP assembly when insect cells were infected with a recombinant baculovirus expressing RVFV structural proteins N, Gn and Gc. In addition, we have also shown evidence of VLP formation when only N and Gc were expressed, in the absence of Gn. Moreover, when RVFV N was expressed alone in absence of both glycoproteins, distinct particulate structures were identified that could be isolated from infected cells.

The N nucleoprotein of *Bunyaviridae *members is the major virion component. It is closely associated with viral genomic RNA along with the L polymerase to form helical ribonucleoprotein (RNP) structures. These RNPs can adopt a circular conformation due to the complementary sequences present at the non-coding regions of the viral genome [31-34]. It is interesting to note that when the N protein of Hantaan virus, another member of the *Bunyaviridae *family, was expressed either by baculovirus or vaccinia virus expression systems, linear structures were formed similar to RNPs [35]. To our knowledge there is no previous data for expression of the N protein of RVFV in an insect cell-baculovirus expression system. Our results have shown that complex circular structures could be purified from recombinant baculovirus infected cells expressing RVFV N protein. These structures were about 56 to 78 nm in size and there were no visible surface projections. It has been reported that RVFV N protein forms dimers in the ribonucleoproteins purified from RVFV infected cells [12]. However, our data indicate that N protein could form multimeric complex and assembled into a particulate structure in the absence of genomic RNA.

The fact that large amount of RVFV N protein could be purified from the media of infected cells suggests that this protein might have a pathway for its release independent to the viral proteins Gn, Gc or the viral genome. In some groups of viruses nucleoproteins can be released outside of host cells when expressed in the absence of other viral proteins [35-38].

The assembly of bunyaviruses takes place mainly intracellularly by budding into the Golgi vesicles. Both glycoproteins Gn and Gc are localized in the Golgi apparatus when expressed as a polyprotein. However, it has been shown that when expressed individually Gc was localized to the ER in absence of Gn [39,40], which suggests that Gc reaches the Golgi apparatus by interacting with Gn. There is no consensus motif for Golgi localization of Gn and Gc among bunyaviruses. In the case of RVFV the Gn contains a Golgi retention motif and the Gc contains a ER retention signal. When these proteins were expressed individually, they localized in Golgi and ER apparatus, respectively [19]. Interestingly a fraction of Gn was also detected on the cell surface when the protein was expressed in the absence of Gc [19]. Additionally, it has been reported that RVFV can also bud from the cell membrane [41] indicating that a fraction of a Gn/Gc complex may be present on the surface of infected cells. Recent work has shown that the overexpression of RVFV glycoproteins using alphavirus vectors produced the expression of Gn and Gc on the cell surface [20]. Therefore, detection of baculovirus expressed Gn and Gc on the surface of infected cells in our study was not entirely unexpected.

Expression of RVFV glycoproteins using the baculovirus expression system has been reported before [24,28] but functional analysis of these proteins was not completed. In order to analyze the expression, correct processing, folding, and interaction of Gn/Gc complex the fusion capacity of Gn/Gc proteins was assessed using a cell to cell fusion assay. In bunyaviruses, Gn/Gc mediates virus entry by fusion of viral and cellular membranes after endocytosis of the virons at low pH [21,29]. In our study we showed that exposure of the infected cells to low pH was necessary to induce fusion activity of the recombinant proteins. A large number of syncytia were observed when cells expressing Gn/Gc were exposed to a low pH for only 2 minutes. The receptor(s) and the cellular factors that are utilized by RVFV during natural infection are still unknown, but equivalents appeared to be present at the surface of the insect cell used for Gn/Gc expression. These results suggest that both proteins were correctly expressed and processed in the insect cells.

Further, the simultaneous expression of N and Gn/Gc in insect cells also readily assembled into VLPs, emphasizing that the expressed proteins were correctly processed. These VLPs could be purified from the supernatant. Under EM, structures with spherical shape and projections protruding from the surface, resembling RVFV virus, were detected. The coexpression of N and Gn/Gc produced reasonably uniform particles with spikes that were clearly visible.

Interestingly, when RVFV N and Gc proteins were coexpressed, VLPs with pleomorphic shapes and sizes could also be purified from the supernatant of infected cells. It is important to note that our constructs for expressing Gc included a frame-shifted Gn ORF. As a result, a peptide of 47 amino acids corresponding to the N-terminal part of Gn would be expressed. The effect of this fragment on the assembly of N/Gc VLPs, if any, was not investigated. In the vaccinia virus expression system approximately half of the total RVFV Gc protein was produced independently from the five AUGs located at the pre-glycoprotein coding sequence [25], most probably due to an internal translation initiation. If this is the case, a fragment of Gn may be expressed. Whether a potentially truncated Gn was expressed in our system which may be functional and supportive to the transport of N/Gc VLPs remains unanswered. Thus our data suggests that even if the truncated Gn might have aided in the production and release of some sort of VLPs, the full-length Gn protein together with Gc, is required for the stable morphology and the spike structures.

In mammalian cells RVFV virus particles are released to the vacuoles of Golgi or endoplasmic reticular sources [7,41]. Our experiment showed that in the baculovirus expression system, the mature VLPs in the vacuoles of insect cells and a large amount of viral inclusion body were also detected in the cytoplasm. It needs further investigation to understand the property and function of these structures in the viral particle formation.

This is the first example of *Bunyaviridae *VLPs that are efficiently generated in a baculovirus expression system. Previously, by expression of the M and S segment of Hantaan virus, VLPs were assembled in mammalian cells using recombinant vaccinia virus but were not produced in insect cells with similar recombinant baculovirus [35]. The success of efficiently producing RVFV VLPs in insect cells and successfully recovering the VLPs from the culture media, together with the finding that the Gn and Gc proteins produced in recombinant Vaccinia virus and recombinant baculovirus efficiently trigger immune reactions in mice to lethal RVFV infections [22,24] indicate that the baculovirus-insect cells is a powerful system to produce large amount of RVFV VLPs for the purpose of vaccine production.

## Conclusion

We have expressed three structural proteins of RVFV either singly or together; the nucleocapsid N protein and the two structural glycoproteins Gn and Gc. The N protein when expressed singly under the control of the polyhedrin promoter was very high level and could be isolated from the supernatant of infected cells. The purified protein formed multimeric complexes and exhibited as a nucleocapsid-like particle (NLPs) structures. When the three proteins were expressed simultaneously by a single recombinant virus, both the Gn and Gc glycoproteins were detected not only in the cytoplasm but also in the cell surface of the infected cells. Expression of these proteins induced cell-cell fusion upon low pH shift. Moreover, VLPs were detected in the cytoplasm and, when purified from supernatant of infected cells, these particles exhibited enveloped structures similar to that of the wild-type RVFV virion particles. Interestingly, Gc and N also formed VLPs with clear spiky structures when they were expressed in the absence of Gn protein. These particles appeared to be more pleomorphic than the VLPs with both glycoproteins, suggesting that both Gn and Gc are needed to generate uniform, stable particles. However, it is clear that Gc and probably also Gn interacts with N protein complex independent of each other. Our results indicate that baculovirus expression system has enormous potential to produce large amount of VLPs that may be used both for fundamental research such as virus entry and morphology study, as well as for vaccination purposes.

## Methods

### Cells and virus

The cell lines used in this study were *Spodoptera frugiperda Sf*9 and *Sf*21. *Sf*9 cells were grown in Sf900II serum-free media (Gibco) and *Sf*21 cells were growth in TC100 media (Sigma) supplemented with 10% fetal calf serum (FCS). Both cell lines were incubated at 28°C. Recombinant baculoviruses based on *Autographa californica *nuclear polyhedrosis virus (AcNPV) were propagated in *Sf*21 cells.

### Source of viral material and antibodies

Purified RVFV viral RNAs were obtained from Dr. Mark Outlaw, National Collection for Pathogenic Viruses, Porton Down, UK. Monoclonal antibodies, against Gn, Gc and N were generously provided by Dr. Connie Schmaljohn (USAMRIID, Frederick, MD). Monoclonal antibodies anti-N, anti-G1, anti-G2, and polyclonal antibody against RVFV virus strain Zinga were provided by Dr. Michele Bouloy (Institut Pasteur, Paris, France). For cell surface expression assay anti mouse-fluorescein isothiocyanate (FITC)-conjugated and anti mouse-tetramethylrhodamine isothiocyanate (TRITC)-conjugated (Sigma) were used. For fusion assay purified anti-baculovirus envelope gp64 protein (e-Bioscience) was used.

### Plasmid construction

The full-length cDNA of the M segment was obtained by reverse PCR using primers 5'-ACGCGTGTCGACACACAAAGATGGTGCATTAAATGTATG-3' and 5'-GAATTCAGATCTACACAAAGACCGGTGCAACTTC-3', and the cDNA of the N protein coding region was generated by reverse PCR using primers 5'-GTCGACGGATCCCCATGGACAACTATCAAGAGCTTCG-3' and 5'-CTCGAGGAATTCAGATCTTAGGCTGCTGTCTTGTAAGCC-3'. The PCR products were cloned into pM83B [42] and translation context sequences were added by site-directed mutagenesis before the 4th ATG for the Gn/Gc with primer 5'-GGTCTTCCATGGCGGCCGCCCGGGCTG CATCCAAC-3', or before the start codon of the N protein with primer 5'-GTTGTCCATGGCGGCCGCGTCGACCTGCAG-3'. The fragment containing the N ORF and the context was transferred to the transfer vector pRN16 (generated in Roy's lab, unpublished), derived from CL29 [43], to produce pRN-N. The fragment including the context and the sequence from the fourth ATG to the end of the Gn was inserted to pRN16 to obtain pRN-4th Gn/Gc. The EcoRV-KpnI fragment of pRN-4th Gn/Gc, which contained the polyhedron promoter and the Gn/Gc genome, was inserted to pRN-N to construct pRN-Ns-4th Gn/Gc. A sequence containing an extra base, C, between the 625^th ^and 626^th ^nucleotides of the M segment was inserted into pRN16 to create pRN-Ns-Gnmut/Gc. This mutation introduces a frame shift after translating 47 amino acid of the Gn and stopped after 8 additional amino acids.

### Expression in insect cells

Bacmid BAc10:KO_1629 _[44] DNA was cotransfected with transfer vectors pRN-N, pRN-N-4^th^Gn_mut_/Gc or pRN-N-4^th^Gn/Gc into *Sf*21, to obtain recombinant baculoviruses containing respective expression cassettes. A modified protocol was used to combine the cotransfection and plaque assay, and individual plaques were picked after six days. The recombinant baculoviruses were amplified in *Sf*21 cells and virus stocks were stored at 4°C. Insect cells were infected with the recombinant virus stocks to examine the recombinant protein expression and VLP production.

### SDS-polyacrylamide gel electrophoresis and Western blotting

Protein expression was analyzed by SDS-polyacrylamide (7.5 to 10%) gels (PAGE) [45]. Proteins were either stained with Commassie brilliant blue or transferred to a cellulose nitrate membrane (Schleicher & Schuell) using a semi-dry transfer cell (Bio-Rad) for Western blotting [46]. Monoclonal antibodies against RVFV Gn, Gc or N proteins diluted 1:1000 in 2% (w/v) milk-phosphate buffer (PBS) were incubated with membranes for one hour. The secondary antibody (anti-mouse IgG conjugated with alkaline phosphatase) (Sigma) was diluted 1:10000. The membranes were finally developed with BCIP-NBT substrate (Sigma).

### N and VLP purification

*Sf*9 cells were infected with the recombinant baculovirus expressing RVFV N protein at MOI of 3 and 4 days post-infection, the media were clarified by centrifugation for 20 minutes at 9000 rpm at 4°C. The supernatant was precipitated through a 20% (w/v) sucrose cushion in TNE buffer (100 mM Tris-HCl, pH 7.4; 100 mM NaCl; 1 mM EDTA) by altracentrifugation (SW28 for 2 hours at 25,000 rpm). The pellet was resuspended in 20 mM Tris-HCl pH 8.0 and further purified by size exclusion liquid chromatography (SEC) gel filtration using Superdex 200 HR 10/30 (Amersham Biosciences). Fractions of 0.5 ml were collected and kept at 4°C for further analysis.

For VLP purification *Sf*9 cells were infected with a recombinant baculovirus expressing N and either Gn/Gc polyprotein or Gc for 4 days. Infected cell medium was harvested and after clarification and ultracentrifugation as before, the pellet was resuspended in 20 mM Tris-HCl pH 8.0 and layered on top of step sucrose gradient 20%, 30% and 40% (w/v) [47] and centrifuged for 4 hours at 190000 × g at 4°C. Alternatively, the sample was purified through a potassium tartrate-glycerol gradient [48] by centrifugation for 18 hours in SW 28 rotor at 28,000 rpm. Visual band or fractions of 0.5 ml were collected and analyzed for the presence of RVFV proteins. Positive fractions were diluted with TNE buffer and ultracentrifuged through a sucrose cushion. The pellet was resuspended in TNE buffer and stored at 4°C.

### Cell surface expression of Gn and Gc

*Sf*9 cells were grown in monolayer on glass coverslips and infected with recombinant baculovirus expressing RVFV Gn, Gc and N. 30 hours post-infected cells were washed and incubated for 20 min in 4% (w/v) paraformaldehyde in PBS, followed by an hour in 1% (w/v) BSA in PBS buffer. As primary antibodies anti-G1 or anti-G2 monoclonals were used at 1:100 dilutions. Subsequently, cells were incubated with secondary antibodies fluorescein isothiocyanate (FITC)-conjugated (Sigma) or tetramethylrhodamine isothiocyanate (TRITC)-conjugated (Sigma) prior to examining the samples by Nikon Eclipse TS100 or Zeiss Axiovert 200 M laser-scanning microscope.

### Fusion Assay

*Sf*9 cells were grown in monolayers and then infected with the recombinant baculovirus expressing Gn, Gc and N at MOI of 0.5. At 24 hours post-infection an antibody against baculovirus gp64 was added to the media. After 2 hours the media was replaced with low pH media and incubated further for 2 minutes and then replaced with normal pH medium. Incubation was continued approximately 2 hours until syncytia were visible. As a control, *Sf*9 cells were infected at MOI of 0.5 with a baculovirus expressing Bluetongue virus (BTV) VP2 protein. Syncytia were counted by visual microscopy at 100× magnification.

### Negative staining and Electron microscopy (EM)

A purified sample was spun in a micro-centrifuge at full speed for 10 minutes. An aliquot of the supernatant was placed onto a carbon-coated grid, dried with the edge of a piece of filter paper and stained with a drop of 3% phosphotungstic acid (PTA) pH 6.8 [7]. All samples were examined using a Jeo 1200 EX transmission microscope.

### Thin-section

Cultured *Sf*9 cells were collected by spinning down at 1000 rpm for 2 minutes and washed once with serum-free fresh culture medium. The final cell pellet was fixed with 2% glutaraldehyde in serum-free fresh culture medium and embedded in agar, and cut into smaller cubes. The cubes were embedded in epoxy resin and ultra sections were cut, mounted onto formva-coated grid, and stained with 2% uracil acetate, pH 5.5 [7].

## Authors' contributions

LL carried out construction of recombinant baculoviruses, purification of proteins and VLPs, and EM studies. CC carried out cell surface expression and cell to cell fusion studies. PR contributed in the coordination and design of the study and helped in the writing of the manuscript.
